# Fabrication and photoluminescent properties of Tb^3+^ doped carbon nanodots

**DOI:** 10.1038/s41598-018-34683-2

**Published:** 2018-11-02

**Authors:** Anna M. Vostrikova, Alina A. Kokorina, Polina A. Demina, Sergei V. German, Marina V. Novoselova, Nadezda V. Tarakina, Gleb B. Sukhorukov, Irina Y. Goryacheva

**Affiliations:** 1 Saratov State University, 83 Astrakhanskaya Street, Saratov, 410012 Russia; 2 School of Engineering and Materials Science, Queen Mary University of London, Mile End Road, London, E1 4NS UK; 3 Skolkovo Institute of Science and Technology, Skolkovo Innovation Center, Moscow, Russia

## Abstract

Carbon nanodots (CNDs) doped with Tb ions were synthesized using different synthetic routes: hydrothermal treatment of a solution containing carbon source (sodium dextran sulfate) and TbCl_3_; mixing of CNDs and TbCl_3_ solutions; freezing-induced loading of Tb and carbon-containing source into pores of CaCO_3_ microparticles followed by hydrothermal treatment. Binding of Tb ions to CNDs (Tb-CND coupling) was confirmed using size-exclusion chromatography and manifested itself through a decrease of the Tb photoluminescence lifetime signal. The shortest Tb photoluminescence lifetime was observed for samples obtained by hydrothermal synthesis of CaCO_3_ microparticles where Tb and carbon source were loaded into pores via the freezing-induced process. The same system displays an increase of Tb photoluminescence via energy transfer with excitation at 320–340 nm. Based on the obtained results, freezing-induced loading of cations into CNDs using porous CaCO_3_ microparticles as reactors is proposed to be a versatile route for the introduction of active components into CNDs. The obtained CNDs with long-lived emission may be used for time-resolved imaging and visualization in living biological samples where time-resolved and long-lived luminescence microscopy is required.

## Introduction

Carbon nanodots (CNDs) are a new class of photoluminescent (PL) labels with distinctive properties that are still challenging to understand. Luminescent CNDs are small (1–4 nm) and often heterogeneous in size and shape. In contrast to organic dyes and semiconductor quantum dots, which have a well-defined organization, the CNDs’ composition and structure are not well understood^1–3^. This lack of understanding makes the effective inclusion of dopants in a CND a challenging task. Carbon-based matrices allow to include in the body of CNDs different atoms and ions, generally to increase PL intensity or modify properties: nitrogen^4–6^, sulfur^7,8^, nitrogen and sulfur^9–11^, silicon^12^, magnesium^13^ or copper^14^. There are not many experimental techniques that can be used to confirm the efficiency of inclusion of metal ions and their interaction with carbon matrices, since the separation of CNDs and low molecular weight compounds is challenging.

In this work we chose to add Tb ions to CNDs because of the unique spectroscopic characteristics of the former, e.g. long PL lifetime, large Stokes shift, and sharp line-like emission bands arising from parity-forbidden *f*−*f* transitions. Photoluminescence of Tb ions becomes intense by means of an “antenna effect”, when chromophores are coordinated to Tb^15^. Several approaches to add Tb or other lanthanide ions to CNDs have been reported in the literature. For example, in the work of Chen *et al*.^16^ Tb-doped CNDs (CND-Tb) were synthesized by dry carbonization of a citric acid and terbium (III) nitrate mixture followed by dissolving of the obtained material in water. PL spectra of CND-Tb synthesized through this route display features typical for CND PL spectra with no PL peaks typical for Tb ions. The hydrothermal method in which citric acid was used as a carbon precursor and lanthanides (Yb^3+^ or Nd^3+^) as doping ions allowed to obtain spherical nanoparticles with both PL in the visible light region from CNDs and the weak infrared sensitized by energy transfer from CND (donor) to Yb and Nd ions (acceptor)^17^. The decoration of already prepared CNDs with Tb (or another lanthanide) ions results in different effects. The presence of Eu ions, associated with carboxylate moieties on the CND surface, induces CND aggregation and quenching their PL^18^. CNDs decorated with Tb were described by Chen *et al*.^19^, these authors show the decreasing of Tb PL lifetime in the presence of CNDs. Also, CNDs with Tb was used as an energy acceptor for dipicolinic acid detection and by Xu *et al*.^20^ for detection of adenosine 5′-triphosphate as an energy donor in fluorescence resonance energy transfer (FRET). Addition of La, Tb or Eu ions to CNDs causes the appearance of metal ions PL and complete PL quenching of the CNDs, obtained in the multistep procedure in organic phase^21^. So far, no convenient method for proper entrapment of Tb ions within CNDs was described.

**Figure 1:**
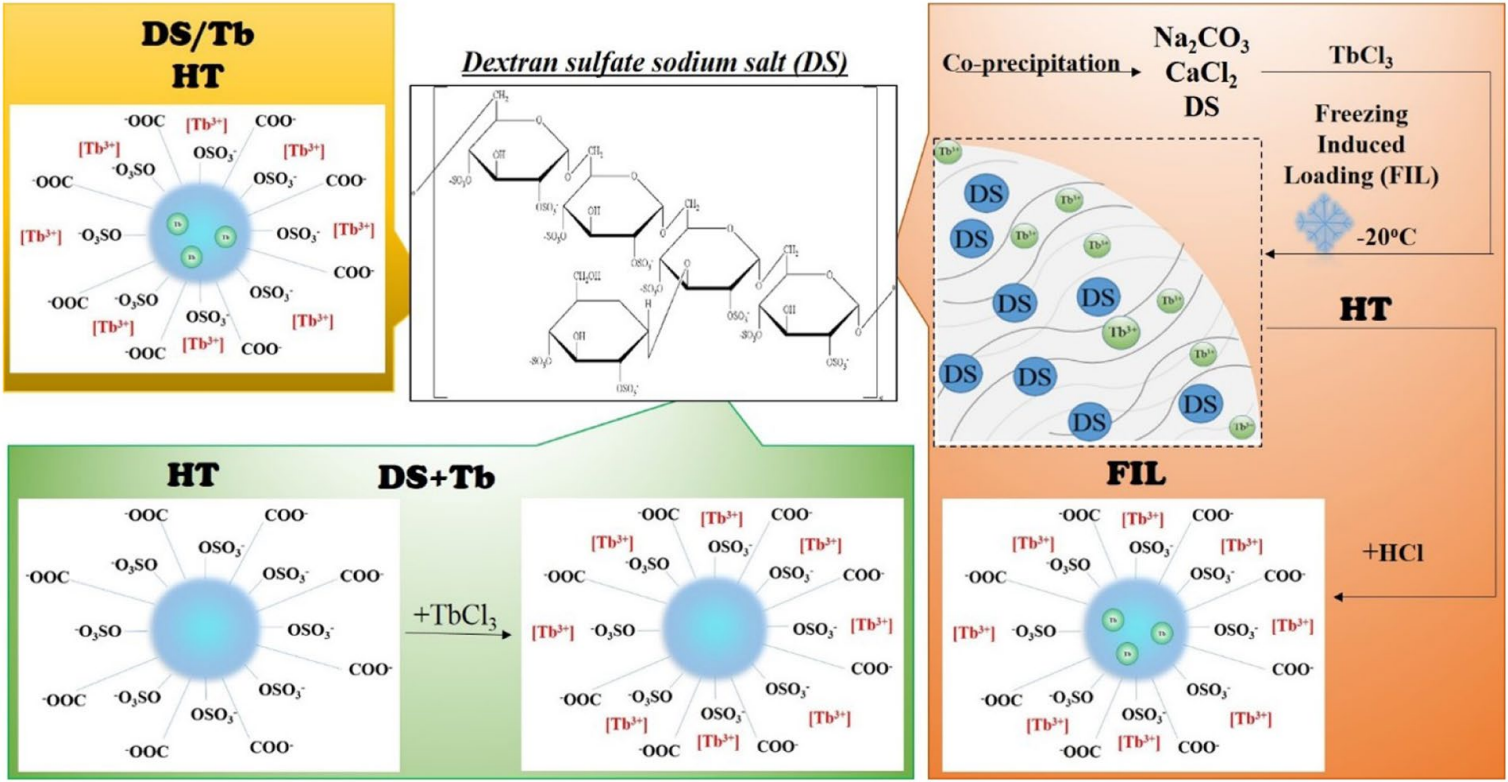
Synthesis of carbon nanodots containing Tb ions (counterclockwise): hydrothermal treatment of CNDs with TbCl_3_ (DS/Tb); CNDs decorated with TbCl_3_ (DS+Tb); freezing-induced loading Tb inside CaCO_3_-DS microparticles (FIL-DS-Tb) with subsequent hydrothermal treatment and CaCO_3_ dissolution.

In this work, we present a new strategy for the attaching of Tb^3+^ ions to CNDs. We explored the possibility of confined geometry synthesis inside pores of CaCO_3_ microparticles and compared the PL properties of the obtained product with properties of a hydrothermally treated mixture of carbon source and terbium salt as well as CNDs decorated with terbium ions (Fig. 1). Sodium dextran sulfate (DS) was chosen as a carbon source due to the natural origin of this polymer and the presence of anionic -COOH and -SO_3_ groups, favoring cation binding with polymer and obtained CNDs. Attachment of Tb^3+^ ions to CNDs is confirmed by changing of Tb PL properties and mobility in conditions of exclusion chromatography.

## Results and Discussion

**Figure 2:**
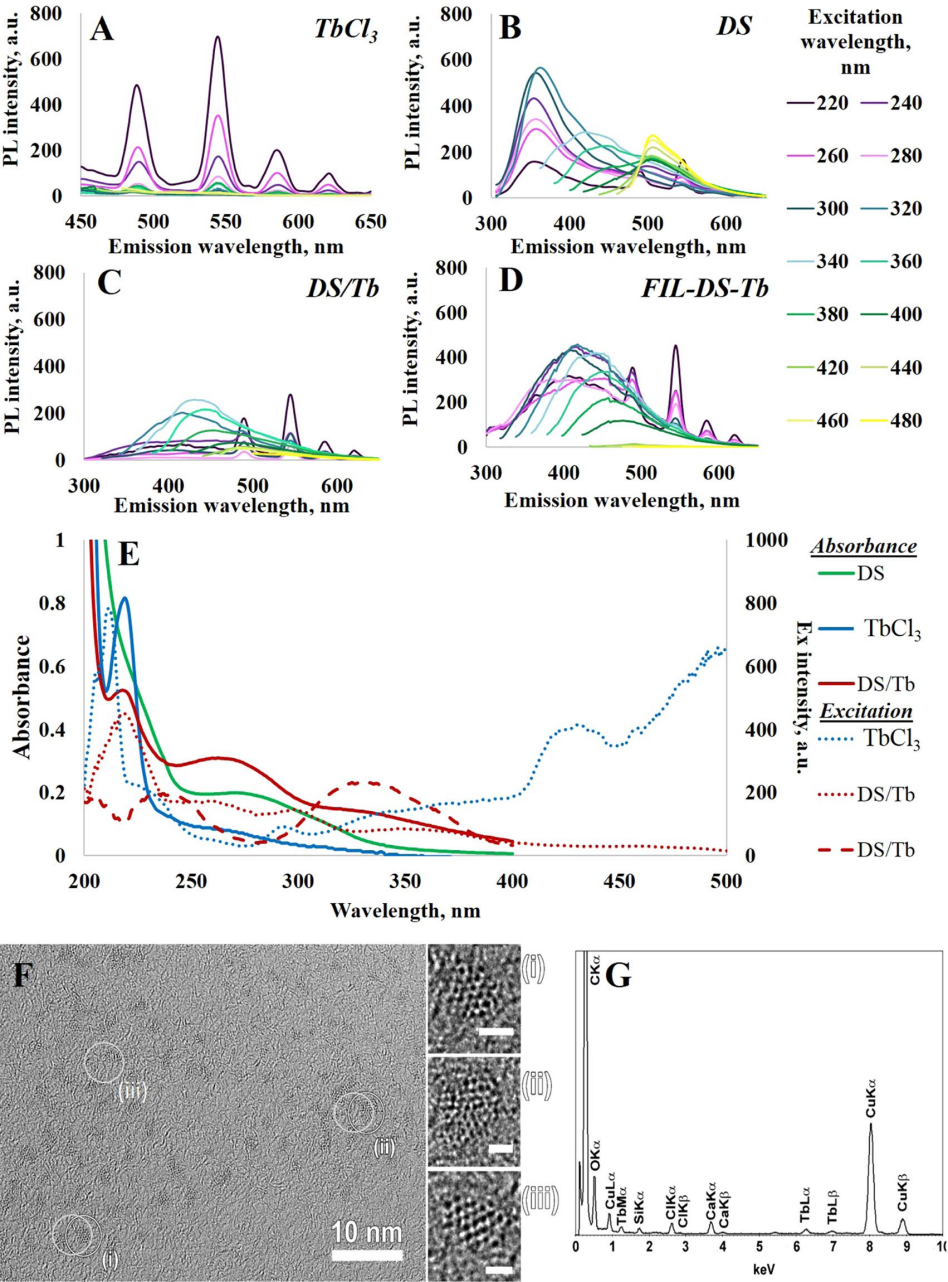
(**A**) Stationary PL spectra of TbCl_3_ solution, (**B**) hydrothermally treated solution of DS and TbCl_3_ (DS/Tb) diluted 10 times (**C**) hydrothermally treated solution of DS with subsequent addition of TbCl_3_ (DS+Tb), diluted 10 times; (**D**) hydrothermally treated CaCO_3_-DS microparticles with freezing-induced loaded Tb, after CaCO_3_ dissolution (FIL-DS-Tb); (**E**) absorbance (solid lines) and excitation (dashed (λ_em_ = 420 nm, related to CNDs) and dotted (λ_em_ = 546 nm, related to Tb ions) lines) spectra for initial DS solution (green), TbCl_3_ solution (blue), hydrothermally treated solution of DS and Tb (DS/Tb) (red). (**F**) HRTEM images of Tb containing nanoparticles, enlargements of the areas marked on the image are shown in (i-iii). Scale bars on (i–iii) correspond to 1 nm. (**G**) EDX spectra of Tb containing nanoparticles. For each experiment here and in the following the TbCl_3_ concentration was 0.023 mg/ml; DS concentration was 7 mg/ml.

As a first and simplest approach hydrothermal treatment of the mixture of carbon source (DS) and TbCl_3_ was tested. DS CNDs with terbium ions (further denoted as DS/Tb) were synthesized at 200 °C for 3 hours^22^. These mild conditions were applied to avoid soot formation: large pieces of carbon soot can sorb terbium ions and reduce the amount of terbium that should be incorporated into CNDs. The PL spectra of DS CND with terbium ions show characteristic CND emission. The obvious terbium PL spectra (characteristic signals of terbium at 490, 546 (strongest), 587 and 621 nm, which are assigned to the ^5^D_4_→^7^F_6_, ^5^D_4_→^7^F_5_, ^5^D_4_→^7^F_4_, and ^5^D_4_→^7^F_3_ transitions) become visible in stationary mode after 10 times dilution of the reaction product (Fig. 2) due to reduction of the photon reabsorption effect^5^. The PL lifetime of Tb ions (0.421 ± 0.005 ms) in TbCl_3_ solution was not sensitive to DS addition (0.417 ± 0.005 ms), but reproducibly decreased after hydrothermal treatment with DS (0.262 ± 0.004 ms). Similar decreasing of Tb PL lifetime was reported by Chen *et al*.^19^ Decrease of the Tb PL lifetime could be an evidence of Tb PL quenching by the structures formed during hydrothermal synthesis. This means the acceptor energy levels of CNDs are less than the energy of the ^5^D_4_ Tb level (20 400 cm^−1^). However, this synthetic route has not resulted in the increasing the intensity of PL bands of CNDs.

As an alternative route for the synthesis of CNDs with terbium ions, we decided to add terbium chloride to the DS CNDs that were previously prepared by DS hydrothermal treatment (further denoted as DS+Tb). From the IR-spectra, one sees that the prepared CND surface has functional groups like -COOH (peaks at 1720 cm^−1^ and 3014 cm^−1^), -C-O-C- (band at 1630 cm^−1^) and –SO_3_ groups from the initial structure of DS (stretching vibrations of the (-S=O) fragment in the area of 1200–1260 cm^−1^ and deformation vibrations at 870 cm^−1^), making it easy for terbium ions to bind with the CND surface. PL spectra of DS+Tb have characteristic Tb signals; the maxima become more intense after 10 times dilution and reduction of the photon reabsorption effect (Fig. 2). The PL lifetime for Tb in DS+Tb (0.264 ± 0.005 ms) was less than in TbCl_3_ solution and comparable with DS/Tb. HRTEM images show the presence of crystalline nanoparticles (Fig. 2F). Energy dispersive X-ray spectra reveal the presence of Tb, Cl, Ca and C (since a Cu grid is used as a TEM support, Cu peaks are present on the spectra as well) (Fig. 2G).

**Figure 3:**
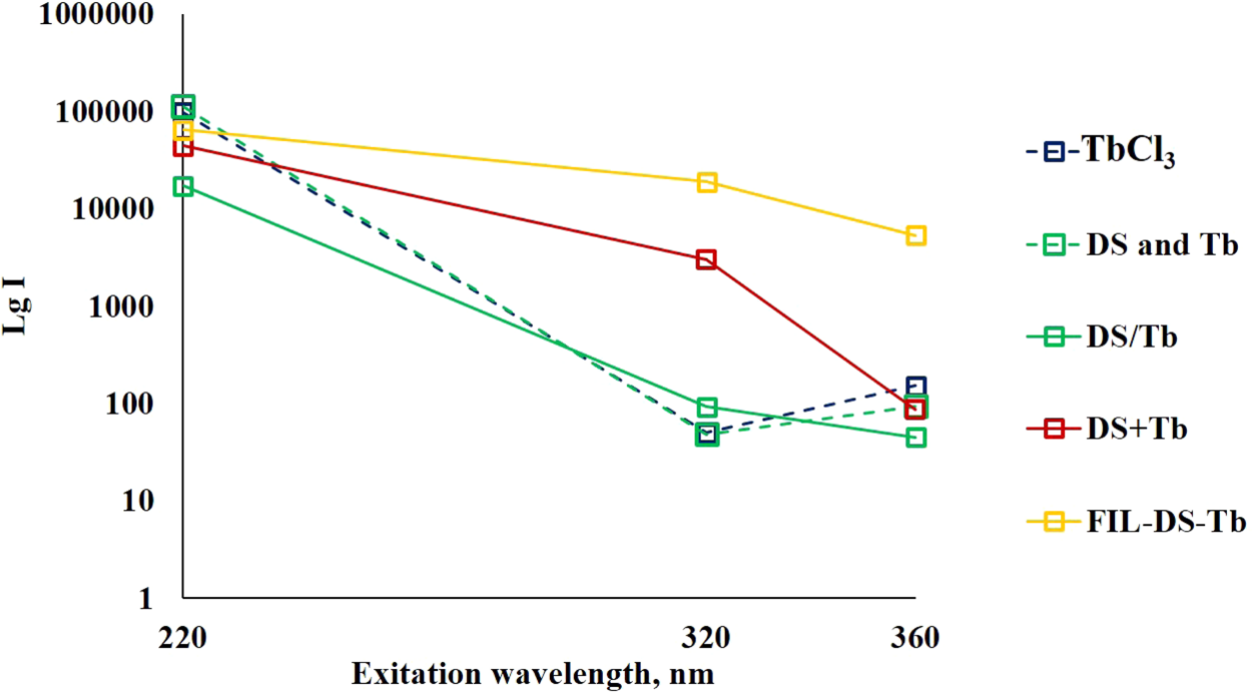
The dependence of lg PL intensity (λ_em_ = 546 nm) from the excitation wavelength in a time-gated mode for TbCl_3_ solution (blue, dotted), solution of DS and TbCl_3_ (green, dotted), hydrothermally treated solution of DS and Tb (DS/Tb, green solid), hydrothermally treated solution of DS with subsequent addition of TbCl_3_ (DS+Tb, red)), and hydrothermally treated CaCO_3_-DS microparticles with freezing-induced loaded Tb, after CaCO_3_ dissolution (FIL-DS-Tb orange).

It is important to note that for TbCl_3_, DS/Tb and DS+Tb solutions terbium PL bands can be excited not only at 220 nm but also at longer wavelengths, although with substantial decreasing of PL intensity at higher wavelengths up to 320 nm (Fig. 3). As can be seen, for CNDs decorated with Tb ions the relative intensity of Tb PL, exited at 320 nm, is higher than for TbCl_3_ and DS/Tb solutions. We speculate that this fact can be the result of the contribution of the energy transfer from CND (energy donor) to terbium ions (energy acceptor).

To prove Tb binding with carbon nanostructures, CND decorated with Tb ions were separated from non-bound Tb ions in solution with gel exclusion chromatography on a Sephadex G-25 gel column^22^. CNDs are very small particles, so it is difficult to separate them from low molecular weight compounds using common approaches, such as filtration, centrifugation or dialysis.

**Figure 4:**
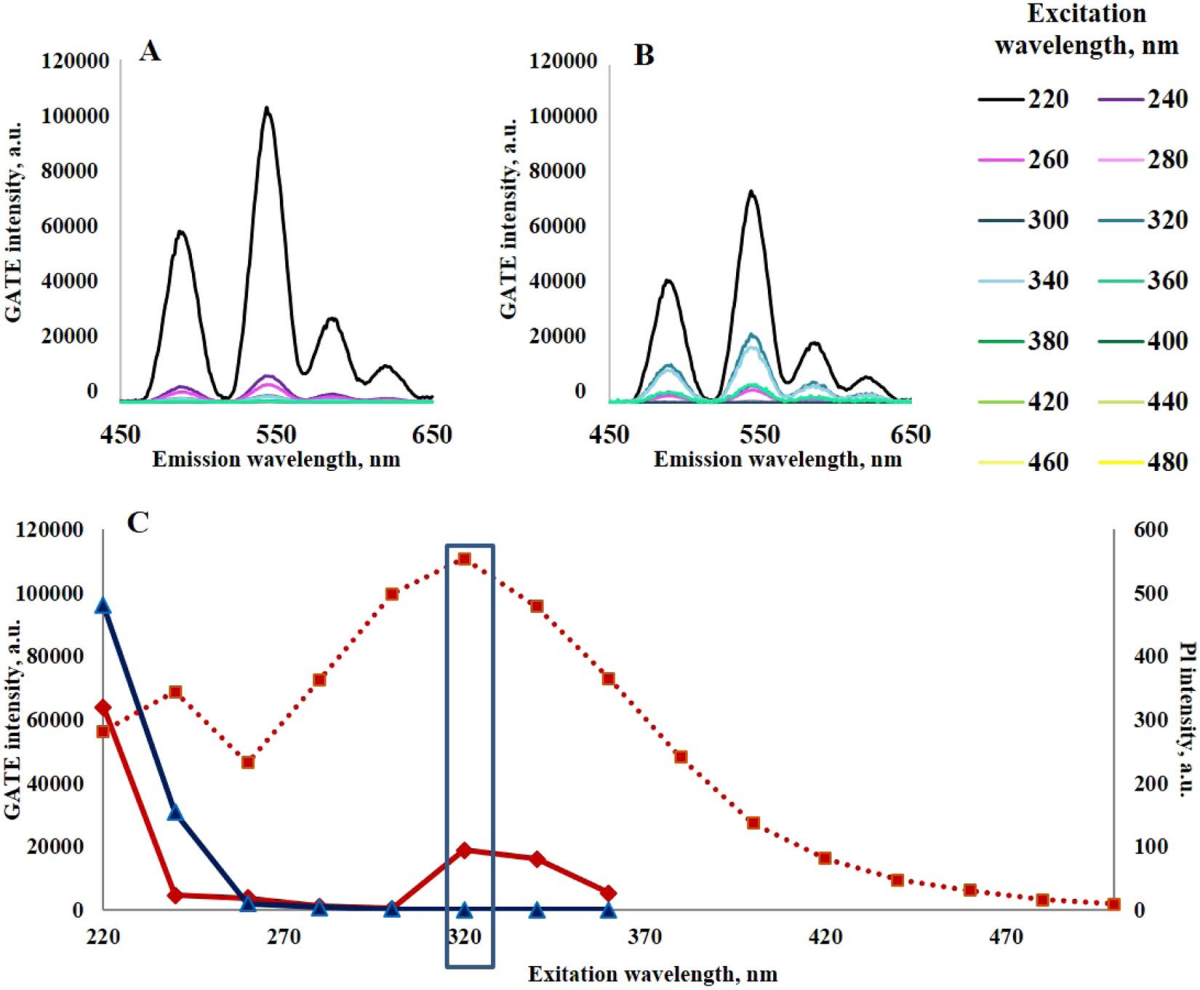
(**A**) Spectra of CNDs, decorated with Tb ions after fractionation with Sephadex G-25 column: absorption spectra of selected fractions; (**B**–**D**) PL spectra of selected fractions with different retention volume: 3.3 ml (**B**) 6.5 ml (**C**) and 10.5 ml (**D**); (**E**) the dependence of PL intensity (λ_ex_ = 220 nm; λ_em_ = 546 nm) of CNDs, decorated with Tb ions (red), and TbCl_3_ (blue) solutions on the retention volume.

To show the dynamics of Tb ions moving through the column, the TbCl_3_ solution was also fractionated. As a result, 70 fractions (totally 50 ml) with different spectral features have been collected for CND DS+Tb and TbCl_3_ solutions. Figure 4 presents absorbance (Fig. 4A) and PL spectra of selected CND fractions (Fig. 4B–D). It is possible to see that there is a clear difference in spectral features. Terbium signals (at 220 nm excitation wavelength) appear in the first fractions (0–4 ml) with high PL intensity (Fig. 4B). Further increase of retention volume up to 6.5 ml leads to terbium luminescent bands excited in a wide range (220–320 nm) and these fractions also have CND signals in the area of 450–500 nm (Fig. 4C,D).

The Fig. 4Е data show a clear difference in Tb ions retention with and without CNDs. The terbium ions from the TbCl_3_ solution leave the column in the fractions with a higher retention volume (6.5–14.5 ml), which corresponds to the retention of low molecular weight compounds. It is worth pointing out that associated with CND Tb ions penetrated through the Sephadex G-25 column in the first fractions with a retention volume of 3.3–6.5 ml. These outcomes show the effective binding of Tb ions with CND. No meaningful signals appeared at a retention volume higher than 25 ml.

As an attempt to further improve interaction, DS and TbCl_3_ were co-precipitated together with inorganic salts (CaCl_2_ and Na_2_CO_3_) in order to obtain CND with Tb ions in the pores of CaCO_3_ microparticles with subsequent dissolution of the obtained CaCO_3_ matrix. Unfortunately, this process was complicated by terbium carbonate precipitation. Tb PL signal has not been shown for the obtained CNDs.

So, a new freezing-induced loading (FIL) technique was developed. The method is based on the loading of dissolved material into a restricted volume of porous CaCO_3_ vaterite microparticles using freezing/thawing process with following the release of loaded material from CaCO_3_ microparticles via dissolving in HCl solution. Two approaches were compared: *(i)* incorporation of Tb ions into pores of СаСО_3_ microparticles with already precipitated DS and *(ii)* incorporation of Tb ions into pores of СаСО_3_ microparticles together with DS (see experimental section). The effectivity of Tb ions FIL was calculated as a ratio of optical density (λ = 220 nm) of Tb-contained solutions after and before FIL procedure. The calculated effectivity of Tb ions FIL into СаСО_3_ microparticles was 78 ± 6% for FIL with the only TbCl_3_ in solution and 1.9 ± 0.1% for FIL of TbCl_3_ together with DS. FIL effectivity of Tb incorporation into СаСО_3_ microparticle pores together with DS was drastically decreased, so DS in solution prevented incorporation of Tb ions. For the future research FIL of TbCl_3_ solution into CaCO_3_ pores already contained DS was used (further denoted as FIL-DS-Tb). After the dissolution of СаСО_3_ microparticles, stationary and time-gated PL spectra were obtained.

**Figure 5:**
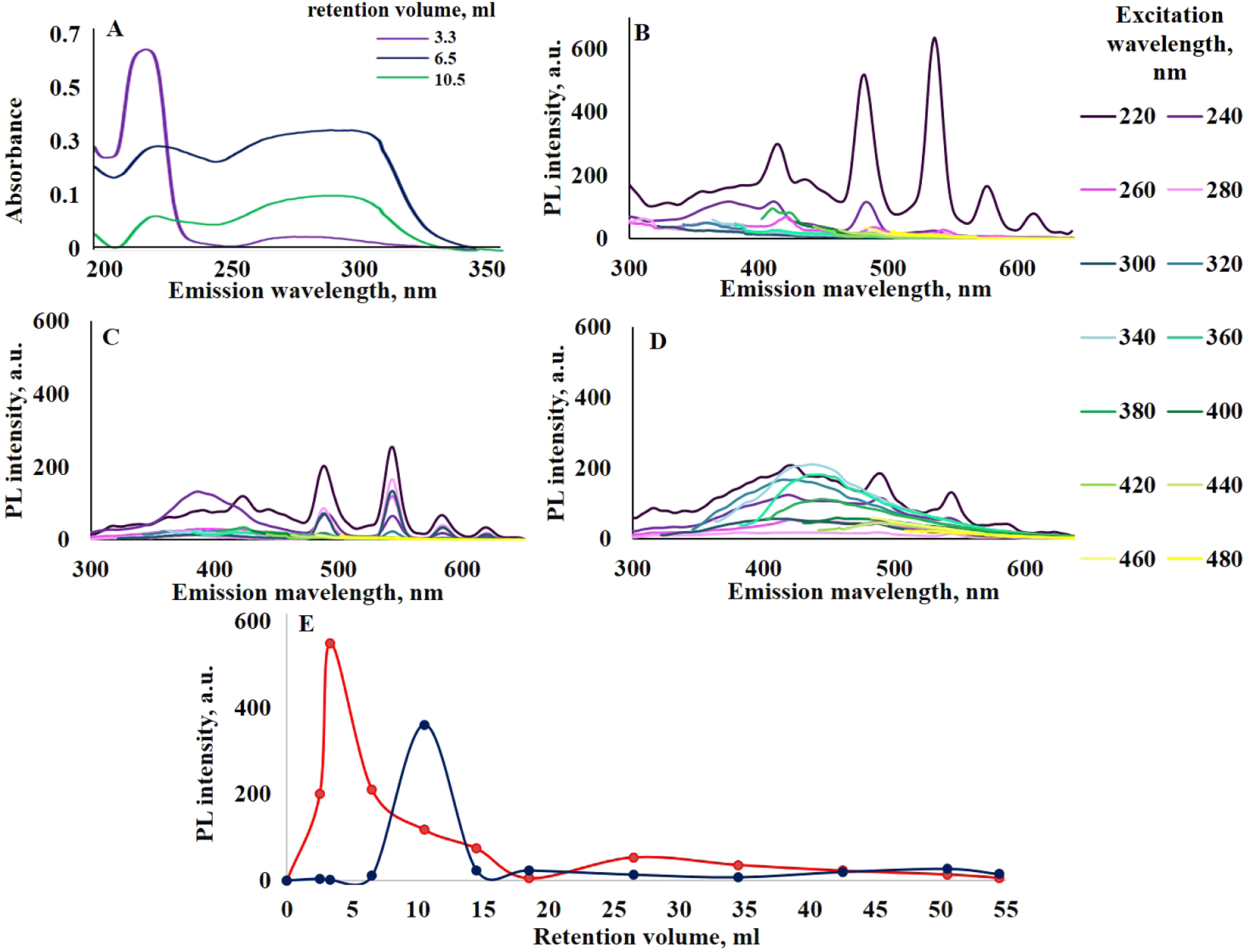
Time-gated PL spectra of solutions: (**A**) TbCl_3_ and (**B**) CNDs obtained *via* freezing-induced loading of TbCl_3_ solution into CaCO_3_ pores already contained DS and follow dissolution of CaCO_3_ (FIL-DS-Tb) solutions. (**C**) Influence of the excitation wavelength on the maximal PL intensity for a TbCl_3_ solution in time-gated mode, emission at 546 nm (blue line); FIL-DS-Tb in time-gated mode, emission at 546 nm (red line) and in stationary mode at a maximal intensity (red dotted line).

Comparison of the time-gated PL spectra of TbCl_3_ (Fig. 5A) and FIL-DS-Tb (Fig. 5B) solutions shows a different dependence of the PL intensity on the excitation wavelength. Figure 5C presents the influence of the excitation wavelength on the Tb PL intensity in the time-gated mode for TbCl_3_ and FIL-DS-Tb solutions, and CND emission intensity at a maximal wavelength for FIL-DS-Tb solution (stationary regime). As can be seen, the PL intensity of TbCl_3_ solution gradually decreases with excitation moving into higher wavelengths. There is no distinguishable Tb PL at the excitation wavelengths longer than 240 nm. In contrast, when CNDs obtained in restricted pore volume were excited at 320–340 nm, we observed characteristic emission bands both from CND (stationary PL) and Tb ions (time-gated mode), as shown in Fig. 4C. The intensity of all four transitions of Tb PL increases. This spectral area coincides with the stationary PL maxima of CNDs. Such matching of profiles could confirm energy transfer from CNDs to Tb ions. The sensitization pathway in luminescent lanthanide complexes generally consists of an initial strong absorption of ultraviolet energy that excites the ligand to the excited singlet (S1) state, followed by an energy migration via intersystem crossing from the S1 state to a ligand triplet (T) state. The energy is non-radiative transferred from the lowest triplet state of the ligand to a resonance state of a coordinated lanthanide ion, which in turn undergoes a multiphoton relaxation and subsequent emission in the visible region^23,24^.

To luminesce, the lowest triplet state energy level of the ligand should be approximately 2000 cm^−1^ higher in energy than the luminescent state of the receiving lanthanide ion, both to fulfill the energetic requirements and to ensure a fast and irreversible energy transfer. Tb^3+^ has suitable energy acceptor levels throughout the 20 500–40 000 cm^−1^ region (as well as ^5^D_4_ Tb level at 20 400 cm^−1^); energy transfer to any of these levels is effective in sensitizing ^5^D_4_→^7^F_J_ transition^25^.

Tb PL lifetime in CND, obtained *via* freezing-induced loading of TbCl_3_ solution into CaCO_3_ pores already contained DS, is 0.206 ± 0.004 ms. This is shorter than for a TbCl_3_ solution and for the all previously described systems. This decrease indicates an evidence of the Tb excited state more effective quenching by CNDs.

## Conclusion

Different synthetic routes have been explored to synthesize carbon nanodots (CNDs) doped with Tb ions. This includes hydrothermal treatment of a solution containing carbon source and TbCl_3_; mixing of CNDs and TbCl_3_ solutions; freezing-induced loading Tb and carbon source inside pores of CaCO_3_ microparticles followed by HT and CaCO_3_ dissolution. Binding of Tb ions to CNDs was confirmed using size-exclusion chromatography and manifested itself both in Tb PL lifetime decreasing and Tb PL intensity increasing.

For all studied CNDs a decrease of Tb PL lifetime was observed (Table 1). The shortest Tb PL lifetime, confirming the most effective Tb-CND coupling was observed in the system exploiting freezing-induced loading Tb together with carbon source inside pores of CaCO_3_ microparticles. The highest increase of Tb PL with excitation at 320–340 nm was also shown for that system (Table 1). That gives us evidence of the most effective interaction CND-Tb while HT occurs in the restricted volume of pores. Thus, freezing-induced loading of cations into CNDs using CaCO_3_ microparticles is suggested as a prospective approach for the induction of active components in CND.

**Table 1:** Characteristics of Tb photoluminescence.

System	Terbium PL lifetime, ms	Ratio of intensity of terbium time-gated PL (λ_em_ = 546 nm), excited at 320 and 220 nm
TbCl_3_ solution	0.421 ± 0.005	0.00051
Solution containing DS and Tb	0.417 ± 0.005	0.00043
CNDs obtained *via* hydrothermal treatment of solution containing DS and Tb (DS/Tb)	0.262 ± 0.004	0.0054
CNDs decorated with TbCl_3_ (DS+Tb)	0.264 ± 0.005	0.068
CNDs obtained *via* freezing-induced loading of TbCl_3_ solution into CaCO_3_ pores already contained DS and follow dissolution of CaCO_3_ (FIL-DS-Tb)	0.206 ± 0.004	0.29

The doped CNDs made by HT in pores of easily dissolvable CaCO_3_ microparticles can be used for making various labels and conjugates with biomacromolecules. Such systems are envisaged for foreseen research on imaging and visualization in living biological samples where time-resolved and long-lived luminescence microscopy is required^26^; short-lived background fluorescence and scattered light are gated out allowing the long-lived PL to be selectively imaged. Thus, CNDs could serve for both purposes: as a scaffold for coordination with Tb^3+^ and as a fluorescence reference in ratiometric nanoprobes. The opportunity to detect signal in ratiometric format is related with two-dimensional signals (PL of CND and time-gated PL of Tb ions) that gives further use of dopant CND.

The proposed approach can be broadened for other cations of interest. Freezing-induced loading inside CaCO_3_ microparticles allows to avoid precipitation of insoluble carbonates (carbonate salts of most metals are insoluble in water). Presence of sulfate (from DS) and carboxyl (as result of DS hydrothermal treatment) favor cations binding with CNDs.

## Experimental Section

### Materials and instruments

Dextran sulfate sodium salt (DS, Mw~40 kDa) was purchased from Sigma Aldrich. Terbium chloride hexahydrate (TbCl_3_·6H_2_O) was purchased from Chimmed. For the preparation of CaCO_3_ microparticles, Na_2_CO_3_ (Reakhim) and CaCl_2_ (CaCl_2_*2Н_2_О, Serva) were used. For the fractionation of CNDs and TbCl_3_ solutions desalting columns with Sephadex G-25 medium from GE Healthcare, UK were used. Bidistilled water was used throughout the experiments.

Stationary PL spectra, time-gated PL spectra (0.1–5 ms) and PL lifetime data, as well as excitation spectra, were obtained using a Cary Eclipse fluorometer (Agilent Technologies, Australia). UV-vis absorption spectra were measured with a Shimadzu UV-1800 spectrophotometer (Shimadzu Inc., Kyoto, Japan). FTIR-spectra were obtained with a FSM-1201 FTIR spectrometer in KBr pellets.

Transmission electron microscopy (TEM) was performed on a JEOL ARM 200 F aberration-corrected transmission electron microscope (Jeol, Japan) operated at 80 kV and equipped with a JEOL energy dispersive X-ray (EDX) detector. For TEM studies, as-obtained solutions were drop-cast on an ultrathin carbon film supported on a Cu grid and dried in air.

### Hydrothermal treatment of CNDs with TbCl_3_ (DS/Tb)

The synthetic route includes preparation of water solution (6 ml) with DS 7 mg/ml (0.042 g) and TbCl_3_16 mg/ml (0.138 g of TbCl_3_·6H_2_O). The solution was stirred about 1 min and transferred into a glass cup, placed into a Teflon cup with a cover, put into a stainless steel autoclave and heated at 200 °C for 3 h. The resulting solution was cooled to room temperature.

### Synthesis of CNDs decorated with TbCl_3_ (DS+Tb)

This procedure has similar steps, but TbCl_3_·6H_2_O (0.138 g) was added after cooling of hydrothermally treated (200 °C for 3 h) DS 7 mg/ml solution (6 ml) and then the mixture was stirred for 2 min before analyzing.

### Freezing-induced loading DS and Tb inside CaCO_3_ microparticles

Equivalent volumes (0.615 ml) of 1 M Na_2_CO_3_ and CaCl_2_ solutions were rapidly poured into 2.5 ml of bidistilled water at room temperature and after intense agitation on a magnetic stirrer the precipitate was filtered off, triply washed with bidistilled water, and dried in air. A solution (2 ml) containing 0.014 mg DS and 0.046 mg TbCl_3_·6H_2_O was added to 0.014 g of obtained CaCO_3_ microparticles. Samples were slowly frozen to −20 °С for 2 hours. After the samples were thawed and centrifuged, the supernatant was taken out and the precipitate was dried and subjected to hydrothermal treatment as described above.

### Freezing-induced loading Tb inside CaCO_3_-DS microparticles (FIL-DS-Tb)

Equivalent volumes (0.615 ml) of 1 M Na_2_CO_3_ and CaCl_2_ solutions were rapidly poured into the 2.5 ml of 7 mg/ml DS water solution at room temperature and after intense agitation on a magnetic stirrer the precipitate was filtered off, triply washed with bidistilled water, and dried in air. Solution (2 ml), contained 0.046 mg TbCl_3_·6H_2_O, was added to 0.014 g of obtained CaCO_3_ microparticles. Obtained samples were slowly frozen to −20 °С for 2 hours. After the samples were thawed, centrifuged, the supernatant was taken out and the precipitate was dried and subjected to hydrothermal treatment as described above.

### Fractionation with Sephadex G-25 column

According to manufacturer recommendations, equilibration buffer was removed from the Sephadex G-25 column and 25 ml of double distilled water flew through the column. In the next step 2 ml of water solution of interest and 0.5 ml of double distilled water were added into the column and the first 2.5 ml of solution that leaked from the column were removed. The third step was adding 60 ml of double distilled water in 5 ml portions. We collected 70 CND fractions with a volume of 800 μl.
